# Transcriptome reconstruction and annotation of cynomolgus and African green monkey

**DOI:** 10.1186/1471-2164-15-846

**Published:** 2014-10-03

**Authors:** Albert Lee, Hossein Khiabanian, Jeffrey Kugelman, Oliver Elliott, Elyse Nagle, Guo-Yun Yu, Travis Warren, Gustavo Palacios, Raul Rabadan

**Affiliations:** Department of Biomedical Informatics, Columbia University College of Physicians and Surgeons, New York, New York, NY 10032 USA; Department of Systems Biology, Columbia University College of Physicians and Surgeons, New York, NY 10032 USA; Genomics Division, the U.S. Army Medical Research Institute of Infectious Diseases, Fort Detrick, MD 21702 USA; Molecular and Translational Sciences Divisions, the U.S. Army Medical Research Institute of Infectious Diseases, Fort Detrick, MD 21702 USA; National Center for Biodefense and Infectious Disease, George Mason University, Manassas, VA 20110 USA

**Keywords:** Cynomolgus macaque, *Macaca fascicularis*, African green monkey, *Chlorocebus aethiops*, RNA-seq, Transcriptome, Genomics, Annotation, Database

## Abstract

**Background:**

Non-human primates (NHPs) and humans share major biological mechanisms, functions, and responses due to their close evolutionary relationship and, as such, provide ideal animal models to study human diseases. RNA expression in NHPs provides specific signatures that are informative of disease mechanisms and therapeutic modes of action. Unlike the human transcriptome, the transcriptomes of major NHP animal models are yet to be comprehensively annotated.

**Results:**

In this manuscript, employing deep RNA sequencing of seven tissue samples, we characterize the transcriptomes of two commonly used NHP animal models: Cynomolgus macaque (*Macaca fascicularis*) and African green monkey (*Chlorocebus aethiops*). We present the Multi-Species Annotation (MSA) pipeline that leverages well-annotated primate species and annotates 99.8% of reconstructed transcripts. We elucidate tissue-specific expression profiles and report 13 experimentally validated novel transcripts in these NHP animal models.

**Conclusion:**

We report comprehensively annotated transcriptomes of two non-human primates, which we have made publically available on a customized UCSC Genome Browser interface. The MSA pipeline is also freely available.

**Electronic supplementary material:**

The online version of this article (doi:10.1186/1471-2164-15-846) contains supplementary material, which is available to authorized users.

## Background

Non-human primates (NHPs) have been indispensable animal models for researchers due to their close evolutionary relationship, similar physiology, and overlapping susceptibility to infectious agents [1–3]. The use of NHP animal models has been pivotal in the success of medical breakthroughs, particularly in the development of vaccines and drugs for polio, hepatitis, and AIDS, and generally in developing preventive and therapeutic measures against emerging pathogens and the threat of bioterrorism [4, 5].

Two of the most commonly used NHPs are Cynomolgus macaque (*Macaca fascicularis*, and African green monkey (*Chlorocebus aethiops*), which have long been considered important models for biomedical research and evolutionary studies [3]. There has been an increasing need for these NHPs since the import of the Indian-origin rhesus monkey (*Macaca mulatta*), a traditional animal model, was banned in 1978. Cynomolgus (CM), a close cousin of rhesus macaque (RM), has been used in neuroscience studies and drug safety testing [3, 6, 7]. In studies of Ebola hemorrhagic fever, CM is a better model than the widely used RM, because CM’s symptoms are more similar to those observed in humans [5, 8]. African green monkey (AG), which is known to be resistant to simian immunodeficiency virus, has also been used as a model organism for HIV research [9, 10].

Knowledge of the transcriptome is critical to the study of disease, the immune system, and the regulation of biological processes, and a reference transcriptome provides a starting point for many types of bioinformatic analyses [11, 12]. There have been many efforts to characterize the transcriptomes of CM and AG. Most of these studies were performed using Expressed Sequence Tags (ESTs), Serial Analysis of Gene Expression (SAGE), and microarrays [6, 13]. However, microarrays require a priori knowledge of sequences that are to be studied, and this renders them undesirable for quantitative expression studies or identification of non-human-specific traits. Thus, for the species that are not well characterized, as with CM and AG, these technologies fall short.

Deep sequencing technologies have significantly increased our ability to generate unbiased genetic data at low costs. The availability of such data has allowed us to make significant progress in understanding the genetic basis of biological processes and systems. The application of deep sequencing techniques to transcriptomics allows the nearly complete characterization of transcriptomic phenomena, such as the description of coding and non-coding RNA expression, the identification of splice isoforms, and the discovery of gene fusions [14–16]. These technologies have been applied at low depth of coverage for a *de novo* assembly of CM, which relied heavily on the human reference genome [17].

Recently, draft genome sequences for both CM and AG were published on NCBI’s website [18, 19]. In this study, leveraging the newly available genomes and using deep RNA sequencing, we construct transcriptome assemblies and gene models — predicted genes each coding a family of transcript isoforms — for CM and AG, obviating the need for computationally expensive and less reliable *de novo* transcriptome assembly [20, 21]. We present the Multi-Species Annotation (MSA) pipeline [22], annotate these new transcriptomes, and assign HUGO standard gene symbols to the gene models. We also identify 13 novel transcripts specific to these species, and elucidate tissue-specific expression profiles among these NHP animal models compared to those of other primates and humans. These transcriptomes are publically available on a customized UCSC Genome Browser interface [23] for users to navigate through the transcriptomes, search for genes of interest, and compare tissue-specific splice isoforms.

## Results

### Transcriptome assembly

To characterize the transcriptomes of Cynomolgus macaques and African green monkey, we generated RNA-seq data for tissue samples from liver, lymph node, lung, spleen, blood (five replicates), marrow (CM only), and brain (AG only) of healthy individuals (see Additional file 1). After filtering the low quality sequences and trimming of low quality bases, we mapped the appropriate sequence reads to the corresponding draft genomes of CM (GenBank Assembly ID GCA_000364345.1) and AG (GenBank Assembly ID GCA_000409795.1) and assembled them using the Tuxedo suite [24]. Non-blood tissue samples were assembled independently using Cufflinks without prior annotations, and merged by Cuffmerge to generate the first draft assemblies. Guided by the first draft assemblies, blood replicate samples were individually assembled by Cufflinks and merged with the previous assemblies (see Methods). This step augmented the draft assemblies yielding 106,570 and 118,896 contig transcripts for CM and AG, respectively (Table 1).

**Table 1:** **The number of contig transcripts generated by the assembly pipeline, and the numbers of transcripts, gene models, gene symbols, and single-exon isoforms after annotation and identification of isoforms in Cynomolgus and African green monkey**

	CM	AG
Contig transcripts	106,571	118,896
Contig transcripts per gene model	4.032	3.418
Multi exonic contig transcripts	91,029	91,759
Single-exon contig transcripts	15,541	27,137
Transcripts (finalized transcriptome)	85,175	89,290
Gene models	19,850	22,543
Unique gene symbols	16,423	17,581
Gene models sharing gene symbols with Human Ensembl 73	16,889	19,125
Single-exon isoforms	3,822	5,251
Genes with single-exon isoforms	3,399	4,703

### Gene annotation and benchmarking

**Figure 1:**
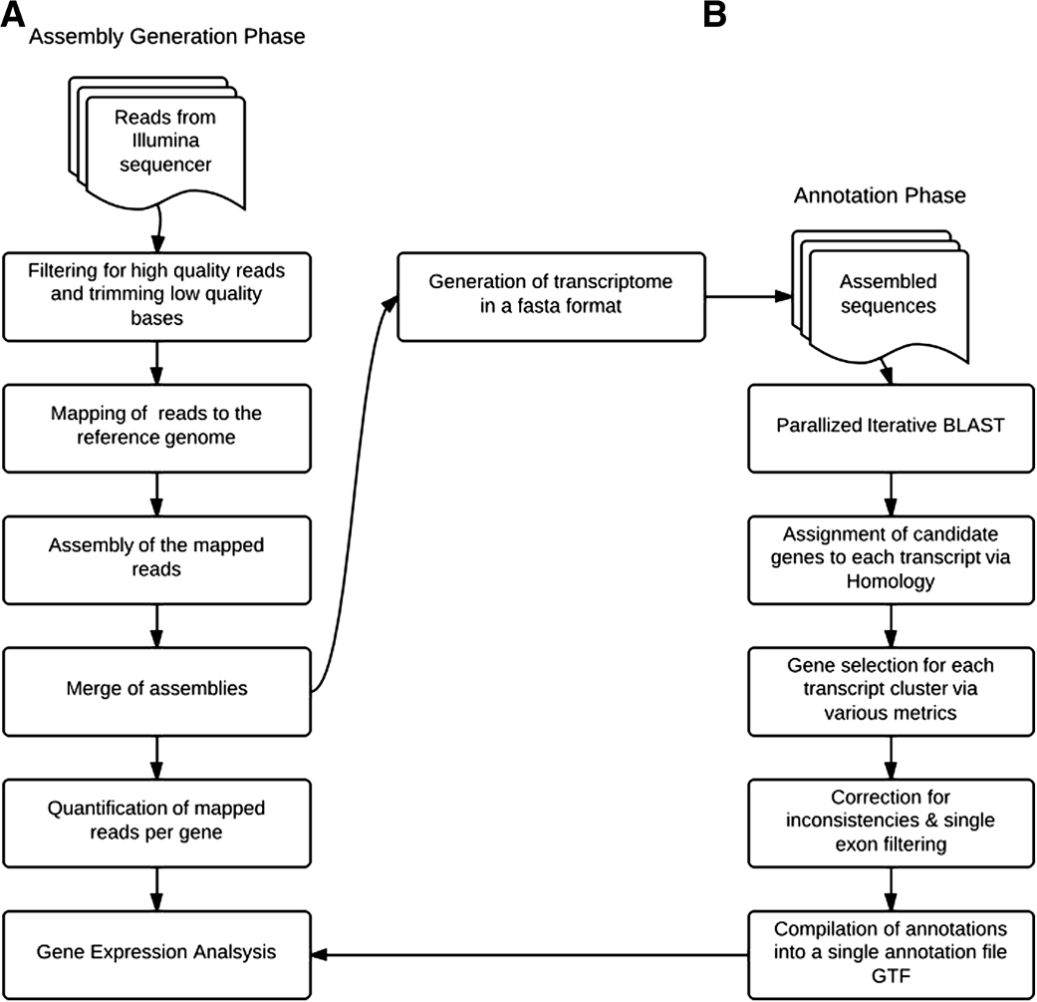
**The schematic of the strategy for reconstructing and annotating transcriptomes.** We leveraged the Tuxedo suite to generate reference-based transcriptome assemblies and used our Multi-Species Annotation (MSA) pipeline for annotation: **A)** We applied TopHat and Cufflinks to quality-filtered, tissue-specific RNA-seq samples, and generated a combined transcriptome assembly using Cuffmerge. **B)** We used the MSA pipeline for annotation. This pipeline has three steps: 1) alignment via parallelized iterative BLAST, 2) assignment of gene symbols via homology, and 3) correction and filtering.

**Figure 2:**
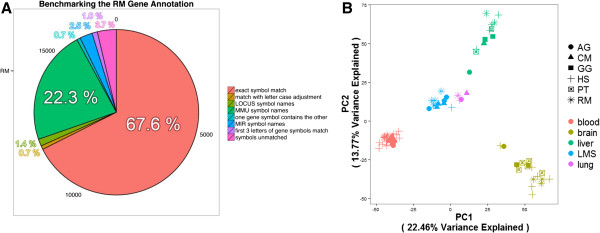
**Assessment of annotations. A)** We benchmarked the MSA pipeline using Ensembl RheMac2 reference transcripts. We annotated 98.4% of the transcripts in RheMac2. 67.6% had unique annotation, matching the assigned gene in the database. The remaining 28.7% of the transcripts were not unambiguously matched due to mis-annotation in Ensembl or inconsistent naming conventions. MMU refers to species-specific *Macaca mulatta* transcripts; MIR refers to microRNA; LOCUS refers to Ensembl identifiers without a gene symbol. **B)** We evaluated the quality of annotations by comparing the gene expression profiles among *Chlorocebus aethiops* (AG), *Macaca fascicularis* (CM), *Gorilla gorilla* (GG), *Homo sapiens* (HS), *Pan troglodytes* (PT), and *Rhesus macaque* (RM). We performed principal component analysis on the expression of 6,463 orthologous genes in multiple tissues. We grouped together lymph node, marrow, and spleen (LMS), as they comprise the lymphatic system. The first two components explained 36.23% of the variance in the data, indicating the consistency of the CM and AG transcriptomes on a biological level.

To systematically describe uncharacterized transcriptome assemblies, we developed the Multi-Species Annotation (MSA) pipeline (Figure 1), based on BLAST alignments to a full primate database (see Methods). To benchmark the performance of the MSA pipeline, we tested it on the Ensembl RheMac2 reference transcripts (release 73) as a control and compared our results with Ensembl’s existing annotation (Figure 2). We were able to annotate 98.4% of the transcripts, of which 67.6% had a unique annotation and matched Ensembl’s gene. The gene symbols for the remaining 28.7% of the transcripts were ambiguously matched, primarily due to inconsistent naming conventions between NCBI and Ensembl, such as Ensembl’s use of species-specific gene symbols, and its occasional mis-annotation (Figure 2A).

Employing the MSA pipeline, we annotated 99.8% of CM’s contig transcripts, of which 87.8% were mapped to validated reference sequences from GenBank and 9.5% to predicted reference sequences [25]. Similarly, for AG, we annotated 99.8% of the contig transcripts, of which 78.8% were based on validated reference sequences, and 8.5% on predicted reference sequences. 13.9% (CM) and 12.5% (AG) of the contig transcripts aligned to sequences lacking gene annotation. For both species, in less than 3.7% of contig transcripts, the MSA-assigned gene symbols did not agree with those of their parent gene models. These mismatches are not necessarily due to limitations in Cufflinks or the MSA pipeline; they frequently stem from inconsistencies in common naming conventions.

### Identification of genes and their isoforms

Using Cufflinks and Cuffmerge, we initially found 30,637 and 43,611 gene models for CM and AG, respectively. We then excluded erroneous contig transcripts, which failed our filtering criteria (see Methods). As a result, for CM, we identified 85,175 transcripts, of which 3,822 were valid single-exon isoforms. The whole set of transcripts corresponded to 19,850 genes models, annotated with 16,423 unique gene symbols. Similarly, for AG we identified 89,290 transcripts, including 5,251 valid single-exon isoforms, corresponding to 22,543 genes models, annotated with 17,581 unique gene symbols.

### Comparative gene expression profiling

It has been demonstrated that the same organs of the different primate species have similar expression profiles [26]. To assess the quality and accuracy of our annotations of CM and AG on a biological level, we hypothesized that if our annotations were accurate, then the expression profile of tissues in CM and AG would cluster with tissues of other primates, such as human, gorilla, chimpanzee and rhesus. To that end, we employed the public RNA-seq data for blood, brain, liver, lung, lymph, marrow, and spleen of human, gorilla, chimpanzee, and rhesus from multiple sources (see Additional file 2) [26–29].

For human, gorilla, chimpanzee, and rhesus, we mapped the RNA-seq reads to the corresponding Ensembl reference genomes and computed the expression measures using the species-specific Ensembl annotation (release 73). For CM and AG, we used the annotations described in this manuscript. We combined the expressions from multiple species using 6,463 protein-coding genes that are one-to-one orthologous between human, chimpanzee, and rhesus and performed principle component analysis (PCA) using the normalized, batch effect adjusted expression values (see Methods). The first principal component was sufficient to separate the expression profiles by tissue and explain 36.22% of the variance in the data (Figure 2B). Clustering of data by tissues indicated the relative consistency in the MSA annotation; however, further experimental validation is required to ensure its accuracy.

**Figure 3:**
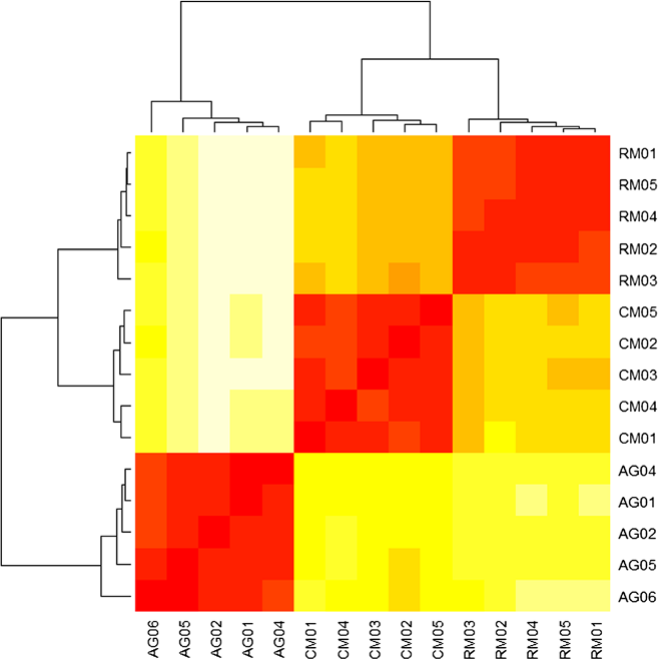
**Reconstruction of the NHP evolutionary tree using expression profiles.** Based on the MSA pipeline’s annotations for 7,927 orthologous genes, we were able to, recover the topology of the evolutionary tree comprising AG, CM, and RM. We used the Pearson correlation as a distance metric between expression values.

To compare CM and AG transcriptomes, we profiled the RNA expression in blood samples for which we had five replicates per species. In this analysis, we also included five replicate blood samples from RM. (We excluded AG03 and CM06 due to the low quality of the samples as shown in Additional file 1). Based on the Pearson correlation metric, we clustered the DESeq-normalized expression values (see Methods) and recovered the topology of the evolutionary tree among these species (Figure 3). This further supported that our annotations were biologically consistent.

### Novel transcripts

**Figure 4:**
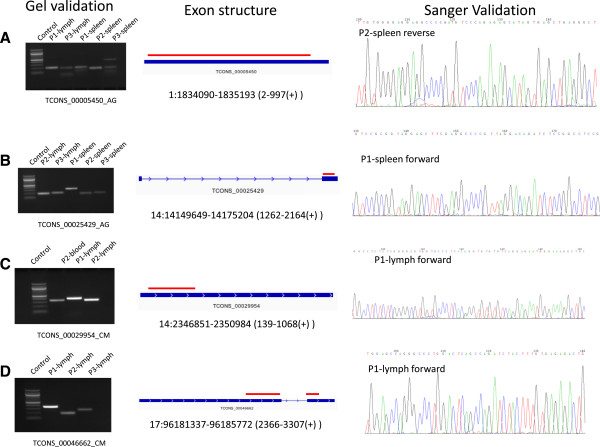
**Validation of novel transcripts.** We validated 11 novel transcripts for CM and 2 for AG. Here, the gel validations, exon structures, and Sanger validations of two novel transcripts from each species is shown (more in Additional file 4). In the exon structure, the red segment indicates the ORF portion of the transcript (structure not scaled proportionally). We used three different primers for each novel transcript to measure expression. **A, B)** Novel transcripts for AG. **C, D)** Novel transcripts for CM.

We sought to determine if there were novel splice isoforms with coding potential in the CM or AG transcriptomes. To that end, we searched for the longest open reading frame (ORF) in each of the six frames, which yielded 1,173,413 and 1,079,987 candidate ORFs for CM and AG, respectively. We translated each ORF sequence into its corresponding protein and aligned it to three protein databases — Refseq, human-nr, and full-nr — in an iterative, subtractive fashion using BLASTP (see Methods). In CM and AG, 10,477 and 16,252 translated ORF sequences, respectively, did not align to any of the proteins in the databases. Among these, 15 transcripts in AG and 26 transcripts in CM had ORFs longer than 300 amino acids (Additional file 3). We selected a subset of these candidate novel transcripts (2 for AG, 11 for CM) for validation in spleen or blood, using two or three different primer pairs. All of the selected candidate novel transcripts were validated with at least one primer in at least one tissue (Figure 4 and Additional file 4).

### Genome browser and release of the data

**Figure 5:**
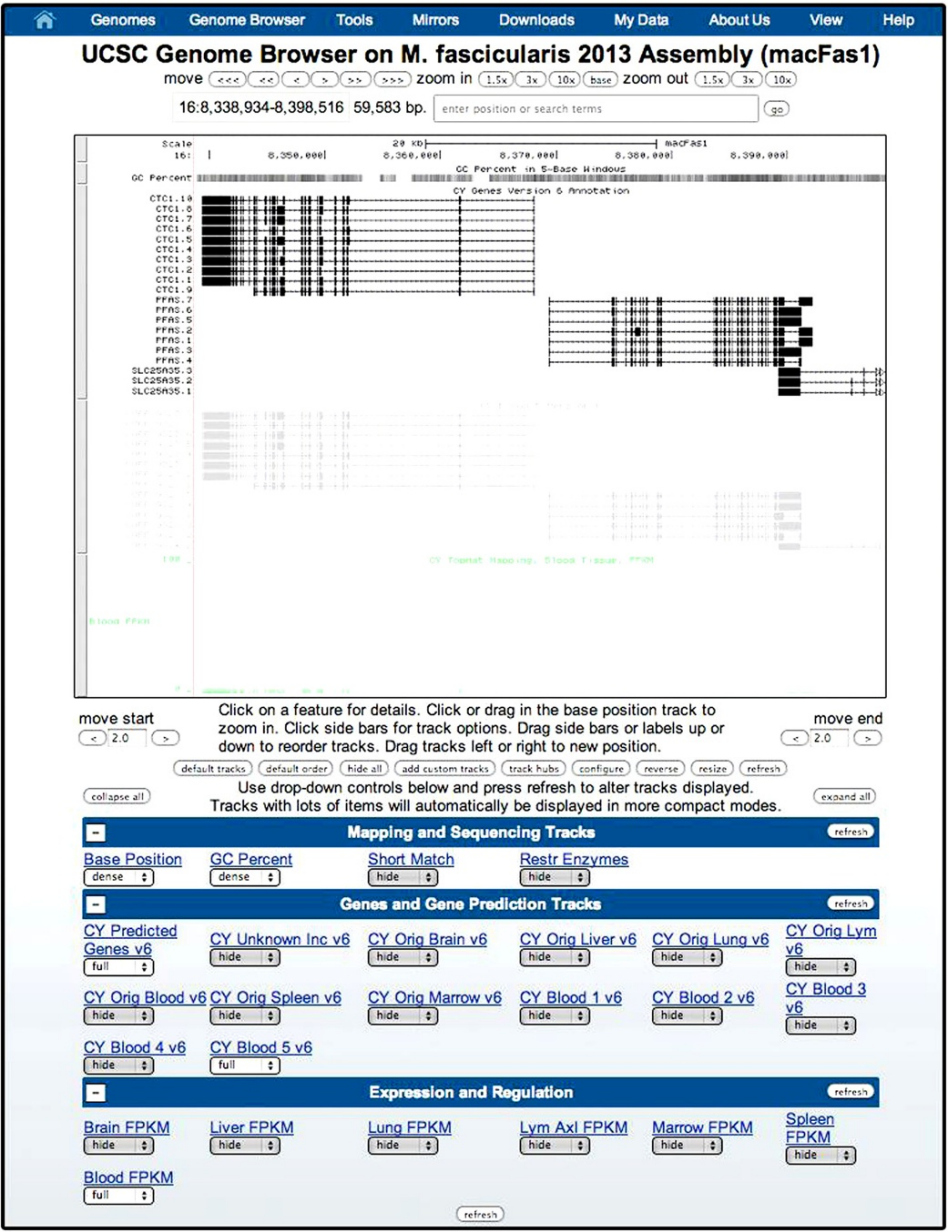
**Browsable annotation of the Cynomolgus and African green monkey on a customized UCSC Genome Browser.** Users can navigate through the transcriptomes, search for genes of interest, and compare tissue-specific splice isoforms on a UCSC Genome Browser on our homepage hosted at Columbia.

The transcriptomes for CM and AG are available on a customized Genome Browser [23], hosted on our server at Columbia University (Figure 5). Through this browser, tissue specific splice isoforms for individual genes in the genomes are accessible.

## Discussion

In this manuscript, we present a draft of the transcriptomes for two important and commonly used NHP animal models: Cynomolgus macaque and African green monkey. We employ deep RNA-seq data from seven tissues and based on the available draft genomes of these species, reconstruct their transcriptomes.

We introduce the Multi-Species Annotation (MSA) pipeline, which annotates assembled contigs and their corresponding gene models through iterative BLAST alignments against a full primate database (Figure 1). This pipeline is novel in that it leverages known sequences of related species to annotate via homology the constructed gene models and their isoforms of an uncharacterized organism with HUGO standard gene symbols. Although we employ the MSA pipeline for AG and CM, it can be used for characterizing transcriptome assemblies from species with evolutionary relatives annotated in the NCBI database. We benchmark the MSA pipeline using Ensembl rhesus macaque (RheMac2) reference transcripts, annotating 98.4% of RM transcripts. This pipeline is freely available for download [22].

We apply the MSA pipeline to the Cynomolgus and African green monkey draft transcriptomes and successfully annotate 99.8% of the contigs. The 19,850 and 22,543 gene models we identify in CM and AG genome, respectively, are in agreement with the expected number of genes in human and primate genomes [26]. The genes in CM and AG are annotated with 16,423 and 17,581 unique gene symbols. Some gene models are annotated with the same symbol due to shared homology.

CM and AG assemblies include 16,889 and 19,125 gene models that share 13,769 and 14,533 gene symbols with the human transcriptome. For these gene models’ isoforms, we compare the distribution of their lengths and their numbers of exons to those in human. The Wilcoxon rank sum test identifies 16,024 and 18,177 gene models (13,059 and 13,827 unique gene symbols) with no significant difference between the two distributions in CM and AG, indicating comprehensive identification of isoforms in these genes. There are 865 and 948 gene models (710 and 706 unique gene symbols) in CM and AG transcriptome, respectively, with significantly different distributions of isoform lengths or exon numbers. These genes may include novel transcripts or in some cases their transcripts may not have been captured in any of the seven tissues in our RNA-seq dataset due to low abundance. The remaining 2,961 and 3,418 gene models (2,654 and 3,048 unique gene symbols) that do not share a gene symbol with any genes in the human transcriptome may also present novel transcripts that require further validation.

We identify about four isoforms per gene on average, 90% and 86% of which contain more than one exon for CM and AG, respectively (see Table 1). Identifying real single-exon transcripts is challenging, as they can be due to artifacts that arise from discrepant or poorly annotated reference genomes [30] or from low read coverage in RNA sequencing [24]. Several studies disregard single-exon transcripts [31, 32]; however, single-exons transcripts have been recognized to play an important role in the evolution of primates [33–35]. The majority of gene models in our genome-based transcriptome assembly pipeline correspond to a single isoform comprising one exon. To eliminate potential single exon artifacts, we focus on genes whose isoforms have no significant difference in the distribution of their length and their number of exons with respect to the human transcriptome, and identify 3,399 and 4,703 gene models with single-exon isoforms in CM and AG, respectively.

To show that CM and AG transcriptomes recapitulate tissue specific expression in human and other NHPs, we compare the expression of the MSA-annotated homologous genes. In both CM and AG, more than 87% of the transcripts are expressed at FPKM values [14] higher than 0.01 in at least one tissue. Focusing on one-to-one orthologous genes among human, chimpanzee, and rhesus (Ensembl 73), the principal component analysis of tissue-specific gene expression in the CM and AG datasets and the publicly available NHP and human datasets shows a similarity in each tissue, indicating the relative consistency of our assemblies and annotations with other primates. We acknowledge that since we relied on draft genome assemblies of CM and AG, our transcriptome assemblies can be improved in quality by obtaining more samples and incorporating the information from *de novo* assembly.

Furthermore, in this study, we report the detection and experimental validation of 13 novel transcripts and splice isoforms with coding potential. Despite the biological importance of noncoding transcripts, we limited our analysis to coding transcripts.

## Conclusion

Overall, the annotated transcriptomes of Cynomolgus macaque and African green monkey presented in this paper will facilitate non-human primate research and improve our understanding of the molecular biology of humans and other primates. As the technology of RNA sequencing improves and more sequences are deposited in public databases, the gene models derived from the RNA-seq and our annotation pipeline will become increasingly accurate.

## Methods

### NHP samples and RNA sequencing

Whole blood samples were harvested from healthy NHPs for rhesus macaque samples RM01 through RM05, African green monkey samples AG01 through AG06, and Cynomolgus macaque samples CM01 through CM06. Blood samples were diluted in 3 to 1 Trizol LS. Tissues were harvested from uninfected NHPs for CM01 and AG01. Bone marrow was unable to be collected from AG01 and brain tissue from CM01. To prepare samples for nucleotide extraction, 0.5 grams of tissue was homogenized in 10 ml of Trizol LS per sample.

RNA was extracted using Trizol LS (Invitrogen, Carlsbad, CA) and used for cDNA synthesis by TruSeq RNA Sample Prep Kit v2 (Illumina, San Diego, CA), according to the manufacturer’s protocol. The libraries were evaluated for quality using the Agilent 2100 Bioanalyzer (Agilent, Santa Clara, CA). After quantification by real-time PCR with the KAPA qPCR Kit (Kapa Biosystems, Woburn, MA), libraries were diluted to 10 nM. Cluster amplification was performed on the Illumina cBot and libraries were sequenced on the Illumina GAIIx using the 76 bp and 100 bp paired-end formats. Additional file 1 describes the details of sequencing results.

### Ethics statement

Each animal received a baseline health assessment, including a complete blood count and blood chemistry, and was determined to be clinically normal on physical examination. All animals were seronegative for measles virus, Macacine Herpesvirus 1, simian immunodeficiency virus, and simian T-cell leukemia virus. All animals were negative for mycobacterium tuberculosis by tuberculin skin test at least 6 months prior to the study. To ensure applicability of results to Animal Biosafety Level 3 and 4 environments, an exemption for partial and/or full contact housing was approved by the IACUC due to the anticipated stress of permanent social separation from a cage mate, the nature of the diseases studied, as well as safety and sanitation concerns. Macaques were singly housed in 4.5-ft^2^ cages with 4 cages per rack (Allentown Caging Equipment, Allentown, NJ), with visual and auditory contact with conspecifics at all times. A form of dietary enrichment was provided once daily. Environmental conditions were maintained as recommended in the Guide for the Care and Use of Laboratory Animals (temperature, 68 to 72°F; relative humidity, 30% to 70%; and 12:12-h light:dark cycle) (18). Animals were fed a commercial primate diet (2050 Teklad Global 20% Protein Primate Diet, Harlan Laboratories, Frederick, MD). Fresh water was chlorinated and filtered at the municipal level (Edstrom Industries, Waterford, WI) and was provided ad libitum. A uniform schedule of food and toy enrichments (Challenge ball, Kong, football, and Dental star, Bio-Serv, Frenchtown, NJ) were used as outlined by our institute’s husbandry and care program.

All research was conducted under an IACUC approved protocol in compliance with the Animal Welfare Act, PHS Policy, and other federal statutes and regulations relating to animals and experiments involving animals. The facility where this research was conducted is accredited by the Association for Assessment and Accreditation of Laboratory Animal Care, International and adheres to the principles stated in the Guide for the Care and Use of Laboratory Animals, National Research Council, 2011(18). Euthanasia was performed to minimize pain and distress by intravenous administration of sodium pentobarbital.

### Genome-based transcriptome reconstruction

To generate high quality assemblies, we first assessed the quality of reads using the FastQC algorithm [36]. We used FASTX-Toolkit to perform trimming, quality filtering, and duplication removal [37]. Additionally, we employed PRINSEQ-Lite [38] to filter transcripts with fewer than 50 bases. For pair-ended libraries, we removed read pairs if both the forward and the reverse (or their complements) were duplicates. We filtered low complexity sequences using the DUST algorithm (threshold 3), and trimmed reads with a quality score of <15 from the 3′-end.

Tophat (version 2.0.8) [24] with default parameters was used to map CM and AG reads to their corresponding reference genomes. In the initial run, the reads obtained from liver, lymph node, lung, marrow, and spleen were mapped to the CM genome; and brain, lymph node, liver, spleen, and lung to the AG genome. Bowtie1 (version 0.12.9) was used as the main aligner for Tophat throughout this study. After the alignment, Cufflinks (version 2.1.1) was used with default parameters to assemble reads into transcripts. Subsequently, the assembled transcripts were merged with Cuffmerge to obtain a non-redundant unified set of transcripts. Blood samples were then added for augmentation and benchmarking of these transcriptomes, via annotation based transcript (RABT) assembly procedure [24, 39] (using --GTF option in Tophat and --GTF-guide in Cufflinks, followed by Cuffmerge).

### Multi-species annotation (MSA) pipeline

We designed the Multi-Species Annotation pipeline to assign gene symbols to contigs through aligning them by BLAST [40, 41] to sequences in NCBI’s nt database. In the present study, we used a cutoff BLAST e-value of 1e-4. For every subject sequence with a hit in the database and corresponding to a unique accession ID, we utilized its Gene Feature Format (GFF) file to describe the coordinates of genes within the sequence. Thus, the pipeline relied on the BLAST output and a concatenated set of GFFs. It is comprised of the following three steps.

The first step was to add information from the GFF files to the BLAST output, as well as to merge local alignments, so there was only one row per unique subject-query ID. This was necessary because BLAST is a local aligner, so pieces of a query sequence can map to multiple locations on a single subject sequence. In this step, we also computed other information, such as query coverage, subject coverage, and gene coverage. To determine which genes were covered, we converted the BLAST results into BED format and used Bedtools-intersect [42] with the coordinates given in the GFF file. This step resulted in a table of every transcript and all the accession numbers and corresponding species to which it mapped, and all the genes with which it intersected.

In the second step, we parsed this table. While the first step merged multiple alignments over unique subject-query IDs, in this step we merged rows across unique query IDs. With this, one transcript pointed to many genes across many different species. (If a gene symbol was not available, only the accession ID was kept.) At this step, some BLAST results can be excluded based on query, subject, or gene coverage information; however, we chose not to apply any of these filters. Gene symbols were canonical-ized into their official HUGO names [43], where possible. Finally, we assigned to each transcript the most frequent gene symbol from the corresponding BLAST alignments to multiple species.

In the final step, we used the Cufflinks gene model prediction, and assigned the consensus gene symbol of all transcript isoforms to the their parent gene model (Additional file 5).

### Identification of isoforms

One gene symbol may annotate multiple gene models in our assembly. We relied on the Cufflinks-predicted position of the gene models and the expression of their contig transcripts in all tissues to filter out erroneous contigs. We excluded contig transcripts with less than 35% cumulative query coverage obtained at the annotation step, which constituted <10% of all transcripts. Then, for each gene symbol, we identified the consensus chromosomal position of all gene models and only included the contig transcripts that matched the position. When there was ambiguity in determining the consensus chromosomal position, we chose the gene model with the highest total expression values, as measured by FPKM [14], in its contig transcripts. At this point, 10-20% of the contig transcripts were predicted to be single-exon isoforms. We limited the identification of single-exon isoforms to the common genes between humans and the NHPs in the study. For each gene symbol, we compared the transcripts’ length distribution and number of exons via the Wilcoxon rank sum test in the human transcriptome (Ensembl 73, excluding processed and nonsense mediated decay transcripts) versus the CM or AG assemblies. The single-exon isoforms in the genes without statistically significant differences in both distributions were then retained and the rest were discarded. We evaluated the use of Wilcoxon rank sum test by applying our methodology to the chimpanzee and RM transcriptomes (Ensembl 73, excluding processed and nonsense mediated decay transcripts). Chimpanzee has 13,841 genes with similar gene symbols to those in human transcriptome and RM has 12,720. However, only 1 gene in chimpanzee and only 46 genes in RM have significantly different transcript length distributions or numbers of exons.

### Gene expression profiling

We obtained public RNA-seq datasets for liver, lymph node, lung, blood, and brain from *Homo sapiens* (HG), *Pan troglodytes* (PT), *Gorilla gorilla* (GG), and *Rhesus macaque* (RM) via Gene Expression Omnibus/ArrayExpress from the following series: GSE30352, E-MTAB-513, GSE52166, GSE50957 [26–29]. We computed the abundance of gene expression using Htseq-count 0.5.3p3 and used DESeq 1.14.0 [44] to normalize for the differences in library size. We used the sva 3.8.0 package [45] in R to adjust for batch effects introduced by combining samples from multiple studies. In particular, we used the ComBat function in the sva package to adjust for batch effects from the five sources of RNA-seq data (USAMRIID, Brawand, Human BodyMap, KirknessSep, and KirknessNov). Since ComBat is designed for microarrays, we converted counts to log-scale and exponentiated the normalized values after normalization. We performed principal component analysis using the prcomp function in R. We obtained the list of one-to-one orthologous genes among human, chimpanzee, and rhesus from BioMart [46]. For Comparative gene expression profiling of blood samples from RM, CM, and AG, we used DESeq-normalized expression values; however, no correction for batch effects was required as these samples were all prepared and processed simultaneously.

### Identification of novel transcripts and validation

We relied on ORFs to define the coding potential for simplicity and ease of analysis. We searched all six frames in each transcript using TransDecoder (rel16JAN2014) [47] and filtered on a minimum amino acid length of 50. We then used BLASTP to iteratively align the translated sequences to the human Refseq proteins, the human subset of the nr protein database, and finally the full nr database (using an e-value cutoff of 1e-2). Compared to BLASTX, this process is computationally efficient and does not compromise sensitivity [48]. We used Primer-BLAST [49] to design primers to validate novel transcripts (Additional file 3).

For validation, RNA was extracted from tissues of Cynomolgus macaques and African green monkeys using Trizol LS (Invitrogen, Carlsbad, CA). cDNA synthesis was performed using the Superscript III First Strand Synthesis System (Invitrogen, Carlsbad, CA). Amplicons were generated with the replicate primer pairs designed for validation using Phusion Hot Start II DNA Polymerase (New England BioLabs, Ipswich, MA) and run on a 2% agarose for confirmation. Positive samples were quantified on the Nanodrop2000 Spectrophotometer (ThermoScientific, Waltham, MA) and Sanger sequenced on the Applied Biosystems 3730xl DNA Analyzer.

## Electronic supplementary material

Additional file 1: **Sample and sequencing information.** The sample information (gender and age) and summary statistics for raw, purged, and mapped RNA-seq reads. (XLSX 14 KB)

Additional file 2: **Samples used for the PCA analysis.** The source of the public and internal RNA-seq data used in PCA analysis. (XLS 42 KB)

Additional file 3: **Novel transcript candidates, ORF sequence information, and primers for CM and AG.** The meta information about the candidate novel transcripts for CM and AG. (XLS 229 KB)

Additional file 4: **Validation of Novel Transcripts.** The validation information on 11 novel transcripts for CM and 2 novel transcripts for AG, with three independent primers. (PPTX 4 MB)

Additional file 5: **Method of determining a gene symbol for a given transcript.** The schematic of the steps in the MSA pipeline for assigning a gene symbol to each transcript. (PPTX 101 KB)
