# Supplementary material for: Transcriptome reconstruction and annotation of cynomolgus and African green monkey
Source: BMC Genomics. 2014 Oct 3;15(1):846. doi: 10.1186/1471-2164-15-846 (PMC4194418; doi:10.1186/1471-2164-15-846)
Supplement: Supplementary file 4 — Additional file 4: Validation of Novel Transcripts. The validation information on 11 novel transcripts for CM and 2 novel transcripts for AG, with three independent primers. (PPTX 4 MB) [file 12864_2014_6521_MOESM4_ESM.pptx]

## Slide 1
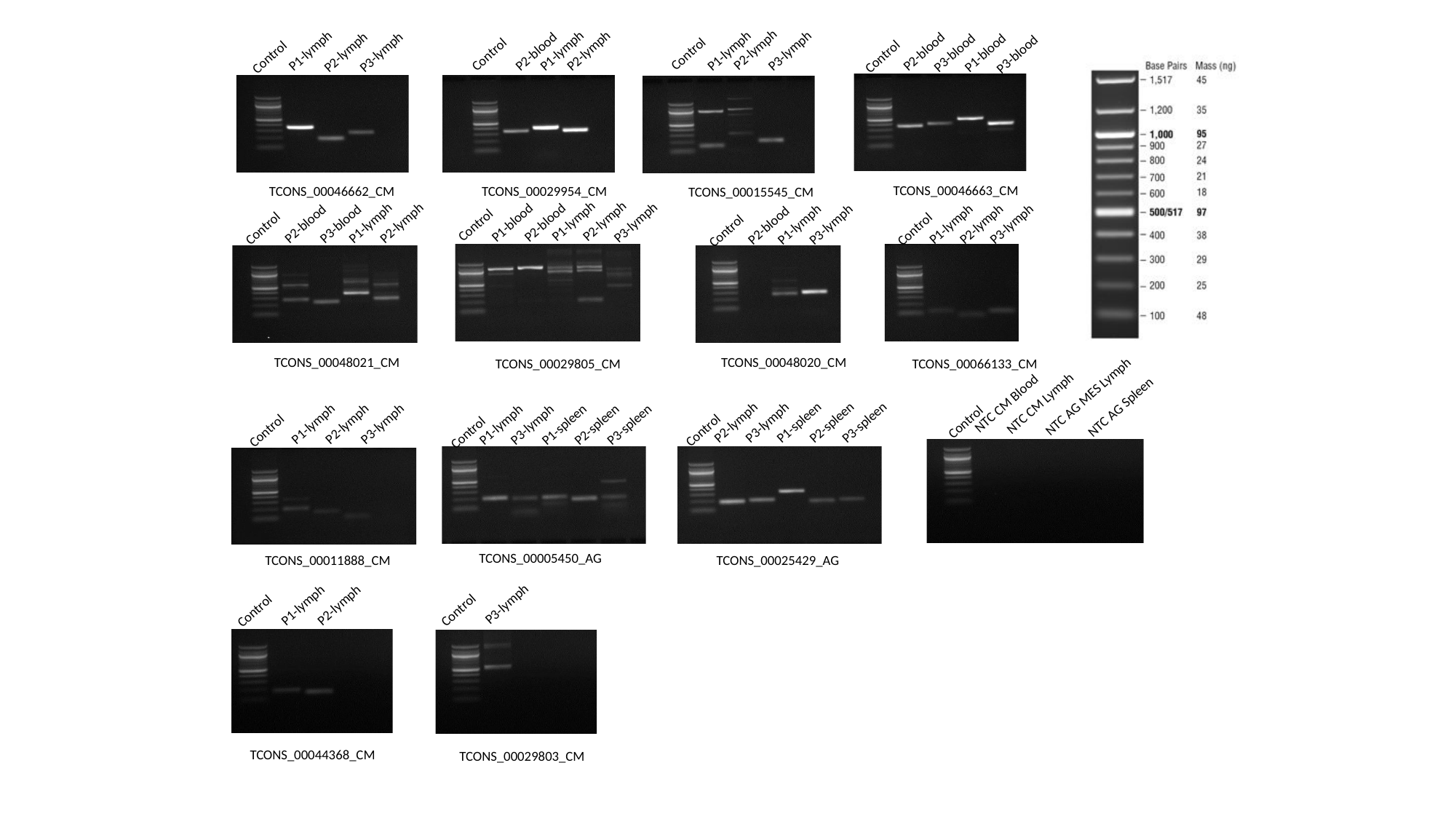

Control
P2-lymph
P1-lymph
P3-lymph
TCONS_00015545_CM
Control
P1-lymph
P2-lymph
P2-blood
TCONS_00029954_CM
Control
P2-blood
P3-blood
P1-blood
P3-blood
TCONS_00046663_CM
Control
P1-lymph
P2-lymph
P3-lymph
TCONS_00046662_CM
Control
P1-lymph
P2-lymph
P1-blood
P2-blood
P3-lymph
TCONS_00029805_CM
Control
P2-lymph
P1-lymph
P2-blood
P3-blood
TCONS_00048021_CM
Control
P1-lymph
P2-lymph
P3-lymph
TCONS_00066133_CM
Control
P1-lymph
P3-lymph
P2-blood
TCONS_00048020_CM
NTC AG MES Lymph
Control
NTC CM Blood
NTC CM Lymph
NTC AG Spleen
Control
P2-lymph
P3-lymph
P1-spleen
P2-spleen
P3-spleen
TCONS_00025429_AG
Control
P1-lymph
P2-lymph
P3-lymph
TCONS_00011888_CM
Control
P1-lymph
P3-lymph
P1-spleen
P2-spleen
P3-spleen
TCONS_00005450_AG
Control
P3-lymph
TCONS_00029803_CM
Control
P1-lymph
P2-lymph
TCONS_00044368_CM
