# Supplementary figures and images for: Transcriptome reconstruction and annotation of cynomolgus and African green monkey
Source: BMC Genomics. 2014 Oct 3;15(1):846. doi: 10.1186/1471-2164-15-846 (PMC4194418; doi:10.1186/1471-2164-15-846)

## Slide 1
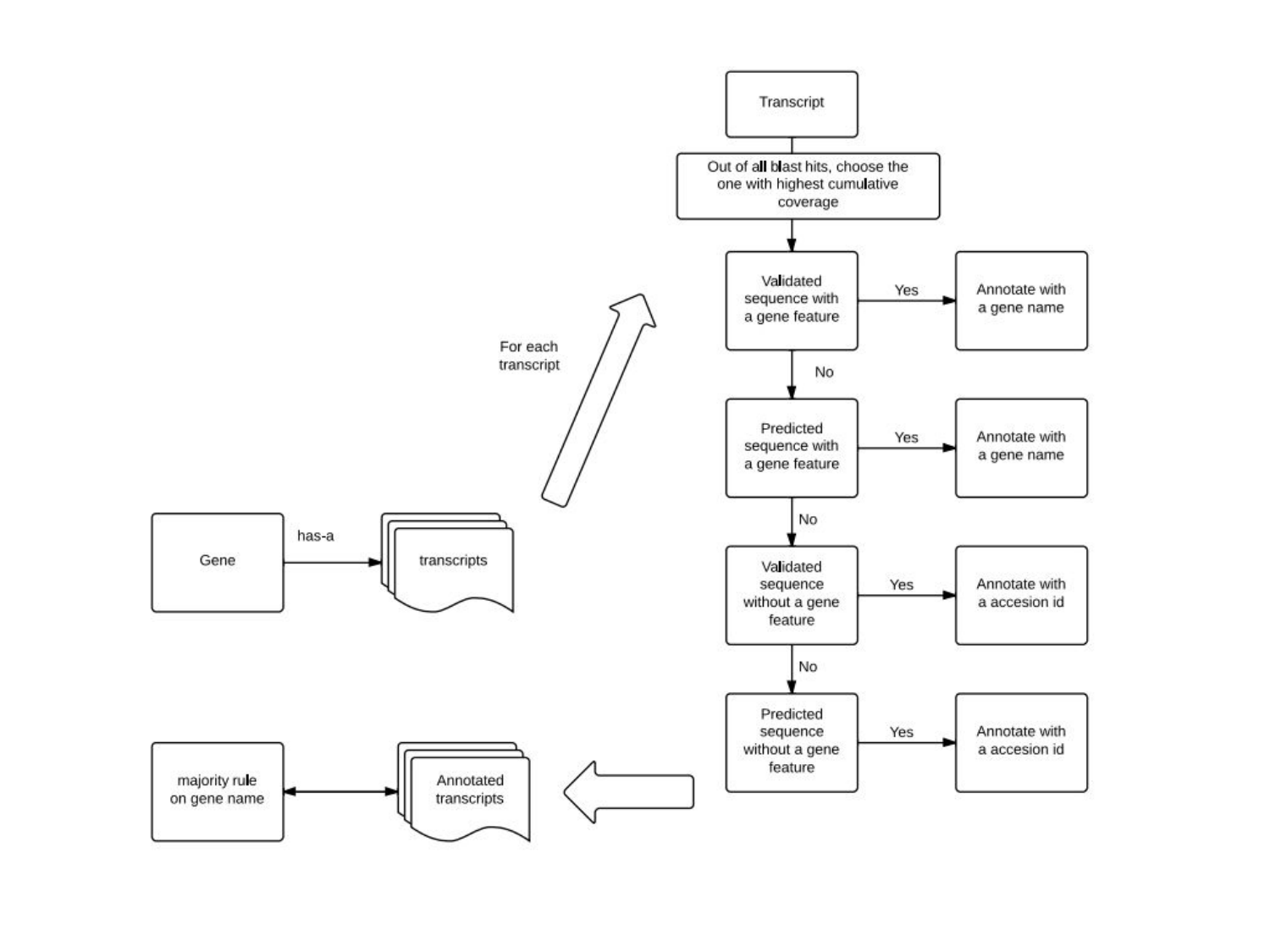

Supplement: Supplementary file 5 — Additional file 5: Method of determining a gene symbol for a given transcript. The schematic of the steps in the MSA pipeline for assigning a gene symbol to each transcript. (PPTX 101 KB) [file 12864_2014_6521_MOESM5_ESM.pptx]
